# Multi-omics analysis of tumor angiogenesis characteristics and potential epigenetic regulation mechanisms in renal clear cell carcinoma

**DOI:** 10.1186/s12964-021-00728-9

**Published:** 2021-03-24

**Authors:** Wenzhong Zheng, Shiqiang Zhang, Huan Guo, Xiaobao Chen, Zhangcheng Huang, Shaoqin Jiang, Mengqiang Li

**Affiliations:** 1 Department of Urology, Fujian Province, Fujian Medical University Union Hospital, Gulou District, 29 Xinquan Road, Fuzhou, 200001 People’s Republic of China; 2 Department of Urology, Kidney and Urology Center, The Seventh Affiliated Hospital, Sun Yat-Sen University, Shenzhen, Guangdong People’s Republic of China; 3 Department of Urology, Shenzhen University General Hospital and Shenzhen University Clinical Medical Academy Center, Shenzhen University, Shenzhen, Guangdong People’s Republic of China

**Keywords:** Tumor angiogenesis, Kidney renal clear cell carcinoma (KIRC), Microenvironment score, Differential methylation sites, Master transcription factors

## Abstract

**Background:**

Tumor angiogenesis, an essential process for cancer proliferation and metastasis, has a critical role in prognostic of kidney renal clear cell carcinoma (KIRC), as well as a target in guiding treatment with antiangiogenic agents. However, tumor angiogenesis subtypes and potential epigenetic regulation mechanisms in KIRC patient remains poorly characterized. System evaluation of angiogenesis subtypes in KIRC patient might help to reveal the mechanisms of KIRC and develop more target treatments for patients.

**Method:**

Ten independent tumor angiogenesis signatures were obtained from molecular signatures database (MSigDB) and gene set variation analysis was performed to calculate the angiogenesis score in silico using the Cancer Genome Atlas (TCGA) KIRC dataset. Tumor angiogenesis subtypes in 539 TCGA-KIRC patients were identified using consensus clustering analysis. The potential regulation mechanisms was studied using gene mutation, copy number variation, and differential methylation analysis (DMA). The master transcription factors (MTF) that cause the difference in tumor angiogenesis signals were completed by transcription factor enrichment analysis.

**Results:**

The angiogenesis score of a prognosis related angiogenesis signature including 189 genes was significantly correlated with immune score, stroma score, hypoxia score, and vascular endothelial growth factor (VEGF) signal score in 539 TCGA KIRC patients. MMRN2, CLEC14A, ACVRL1, EFNB2, and TEK in candidate gene set showed highest correlation coefficient with angiogenesis score in TCGA-KIRC patients. In addition, all of them were associated with overall survival in both TCGA-KIRC and E-MTAB-1980 KIRC data. Clustering analysis based on 183 genes in angiogenesis signature identified two prognosis related angiogenesis subtypes in TCGA KIRC patients. Two clusters also showed different angiogenesis score, immune score, stroma score, hypoxia score, VEGF signal score, and microenvironment score. DMA identified 59,654 differential methylation sites between two clusters and part of these sites were correlated with tumor angiogenesis genes including CDH13, COL4A3, and RHOB. In addition, RFX2, SOX13, and THRA were identified as top three MTF in regulating angiogenesis signature in KIRC patients.

**Conclusion:**

Our study indicate that evaluation the angiogenesis subtypes of KIRC based on angiogenesis signature with 183 genes and potential epigenetic mechanisms may help to develop more target treatments for KIRC patients.

**Video Abstract**

**Supplementary Information:**

The online version contains supplementary material available at 10.1186/s12964-021-00728-9.

## Background

Malignant renal cell carcinoma (RCC) account for 2% of the global cancer burden, and ~ 350,000 new cases will occur worldwide in 2018 [1]. There are several histopathological subtypes of RCC, each characterized by a specific molecular pattern. Kidney renal clear cell carcinoma (KIRC) is the most prevalent heterogeneous subtype, accounting for 75% of all renal cell carcinomas (RCC) and most of them arises from the proximal tubule cells of the renal nephron [2]. The prognosis of metastatic KIRC patients remains poorly, and less than 10% of these cases were alive over 5 years after diagnosis [3, 4]. The development and metastasis of malignant tumor cells depends on the establishment of a sufficient blood supply, that is, tumor angiogenesis. In most normal tissues, factors that anti-angiogenic is predominate, however in frequency dividing organs and tissues, the balance of angiogenesis factor shift to pro-angiogenic growth effect [5]. During the process of angiogenesis, tumor cells express high level of pro-angiogenic growth factor and overwhelming the effects of anti-angiogenic factor to the development of new blood vessels [6].

The initial of tumor angiogenesis program involves a series of change in local equilibrium cells between anti-angiogenic and pro-angiogenic regulators that are produced by surrounding cancer stem cell, stromal cells, infiltrating leukocytes, and cancer cells [5, 7]. Therefore, aberrant angiogenesis in tumorigenesis and metastasis is closely related to abnormal tumor microenvironment. Hypoxia and the expression of the hypoxia inducible factor (HIF) proteins in cancer microenvironment were contributed to tumor angiogenesis and linked to poor prognosis in kidney cancer patients [8–10]. This is easy to state, as tumor cell grows require an adequate supply of oxygen and nutrients based on vascular supply and uncontrolled cancer cell exceed a size that outgrows their host vascular system supply and result in local hypoxia. Consequently, hypoxia and related HIF family proteins such as HIF-α proteins (1α, 2α, and 3α) and HIF-β (1β, 2β, and 3β) proteins stimulate of neoangiogenesis and/or vasculogenesis for cancer cells growth [11, 12]. On the other hand, hypoxia induced release of chemoattractants (such as HIF-1α and HIF-2α) and tumor derived cytokines (such as IL-10, IL-4, and TGF-β) are able to hijack tumor-associated macrophages (TAMs) and tumor-associated dendritic cells (TADCs) functions, resulting in tumor metastasis [13, 14]. In addition, the TAMs in hypoxia microenvironment achieve a pro-angiogenic performance either by upregulating angiogenic molecules (such as VEGF, type I receptor for VEGF, angiopoietin, FGF2, CXCL8, and IL-8) or through upregulation of angiogenic modulators (such as COX2, MMP7, and iNOS) [15]. Therefore, hypoxia, abnormal immune status, and angiogenesis form a positive feedback regulation cycle to promote cancer cells metastasis. In this study, we systematically analyzed the relationship between angiogenesis signals, immune signals, and hypoxia signals in TCGA KIRC patients.

With the breakthrough of targeted therapy, anti-angiogenic targeted therapy has attracted more and more attention in advanced or metastatic renal cell cancer research [8, 16]. Clearly, vascular endothelial growth factor (VEGF) which served as pro-angiogenic growth factor is an effective drug target in metastatic kidney cancer patients and many agents to inhibit this pathway have shown clinical benefit [17, 18]. On the other hand, multi-targeted tyrosine kinase inhibitors (TKI) that inhibit vascular endothelial growth factor receptors (VEGFRs) have been approved by the America food and drug administration (FDA) as standard treatment for advanced or metastatic KIRC patients [19]. Immune checkpoint blocker, such as anti-PD-1 antibody, heralded a new era in the therapy of metastatic renal cell carcinoma (mRCC) [20]. Anti-angiogenic targeted therapy may also enhance the effect immune checkpoint blocker by up-regulating major histocompatibility complex class I (MHC-I) expression and promoting T cell infiltration [20]. Therefore, combining an anti-angiogenic targeted therapy with an immune checkpoint blocker is a closely tailored treatment to test in the clinic. In 2020 National Comprehensive Cancer Network (NCCN) clinical practice guidelines in renal cancer, anti-angiogenic targeted therapy and immunotherapy combination therapies have been added to first-line treatment options [21]. On the other hand, neoadjuvant targeted therapy in both localized and locally advanced RCC has emerged as a strategy to render primary kidney cancer amenable to planned surgical resection in settings where radical nephrectomy (RN) or nephron-sparing surgery (NSS) was not thought to be feasible or safe [22]. Pre-surgical tumor reduction (TR) has been demonstrated in a number of randomized double-blind placebo-controlled studies, and an expanding body of literature suggests clinical benefit in select RCC patients [23, 24]. However, these studies also showed that RCC patients responsiveness to neoadjuvant anti-angiogenic targeted therapy vary from individual to individual [23]. Therefore, evaluation of angiogenesis subtypes in RCC patient might help to reveal the mechanisms of cancer and develop more target treatments for RCC patients.

In this study, we systematically studied the tumor angiogenesis subtypes and potential regulation mechanisms in TCGA-KIRC patients. Briefly, a reference gene set including 183 angiogenesis associated genes was used to generate the angiogenesis score and served as angiogenesis signal in KIRC patients. The angiogenesis score was significantly correlated with immune score, stroma score, hypoxia score, and vascular endothelial growth factor (VEGF) signal score in 530 KIRC patients. Clustering analysis based on 183 genes in reference gene set identified two prognosis related angiogenesis subtypes in KIRC patients. In addition, two clusters also showed different angiogenesis score, immune score, stroma score, hypoxia score, VEGF signal score, and microenvironment score. DMA identified 59,654 differential methylation sites between two clusters and part of these sites were correlated with tumor angiogenesis genes including CDH13, COL4A3, and RHOB. In addition, RFX2, SOX13, and THRA were identified as top three master TFs in regulating angiogenesis signature in KIRC patients.

## Materials and methods

### Data acquisition from public online database

Molecular data of samples pathologic diagnosed with RCC were obtained from The Cancer Genome Atlas (TCGA) database. High-throughput sequencing (HTSeq) transcriptome data of KIRC cohort in TCGA database including 539 RCC samples and 72 tumor adjacent normal samples were directly downloaded from Genomic Data Commons using “TCGAbiolinks” package in R software. The HTSeq transcriptome data (RNA Sequencing) was primary quantified as raw read count expression matrix for differential expression gene analysis. In addition, RNA-seq transcriptome count matrix including 539 RCC samples were further transferred into a transcripts per kilobase million (TPM) matrix, and was then used to calculate the angiogenesis score, microenvironment score, and immune cell infiltration (ICI) profiles of TCGA RCC patients. Corresponding clinical information of TCGA RCC patients was obtained from cBio Cancer Genomics Portal online database (cBioPortal: https://www.cbioportal.org/). An independent RCC data E-MTAB-1980 [25] based on A-MEXP-2183 Agilent Human Gene Expression 4 × 44 K v2 Microarray 026652 G4845A with overall survival information was download from EMBL-EBI database (https://www.ebi.ac.uk/) and served as validation data. TCGA KIRC DNA methylation data which corresponded to the cases with RNA-seq data based on Illumina Infinium Human Methylation 450 K platform including 483 RCC samples were downloaded from California University Santa Cruz Xena Public Data Hubs (https://xenabrowser.net/datapages/). DNA methylation data including 483 RCC samples was presented as a β value matrix. The β value of each probes were calculated by the following equation: β value = Mean_methylated/(Mean_methylated + Unmethylated). 332 RCC cases with somatic mutations (SMs) and 530 RCC cases with somatic copy number variations (CNVs), which corresponded to the RCC samples with RNA-seq data, were obtained from TCGA online database. To calculate the angiogenic score of RCC samples, 10 angiogenic related gene sets (Additional file 4: Table S1) were down from Molecular Signatures Database (MSigDB) (https://www.gsea-msigdb.org/gsea/msigdb).

### Gene set variation analysis

As the strengths of gene set enrichment (GSE) analysis include dimension reduction, noise, and greater biological interpretability. Gene set variation analysis (GSVA) method provides increased power to detect subtle pathway activity changes (such as angiogenic signature) over a sample population in comparison to corresponding methods [26]. In this study, GSVA method was used to calculate the angiogenic value (angiogenic score) of each RCC sample based on the reference gene set obtained from MSigDB. In other words, angiogenic score represent the GSVA value of of each RCC sample. To calculate the angiogenic score of RCC samples, 10 angiogenic related gene sets (Additional file 4: Table S1) were down from Molecular Signatures Database (MSigDB) (https://www.gsea-msigdb.org/gsea/msigdb) [26]. GSVA is an unsupervised, non-parametric method for evaluating the variation of corresponding gene set enrichment through the independent samples of a gene expression matrix. In this study, gene set variation analysis (GSVA) method was used to calculate the angiogenic score each RCC sample based on the reference gene set obtained from MSigDB. GSVA is an unsupervised, non-parametric method for evaluating the variation of corresponding gene set enrichment through the independent samples of a gene expression matrix. The input materials for GSVA method are an expression matrix and a reference gene sets. Specifically, a transcripts per kilobase million (TPM) matrix obtained from TCGA database was served as expression matrix. On the other hand, 10 angiogenic related gene sets were used as reference gene set. In this study we performed “gs*va ()*”function in gsva package, a bioconductor R package, to calculate the angiogenic score, hypoxia score, and VEGF signal score of each RCC sample.

### Immune cell infiltration analysis

In this study we performed ssGSEA algorithm implanted in xCell R package to calculate the enrichment score (ES) of 64 stromal and immune cell types in RCC samples [28]. xCell is a method that integrates the advantages of gene set enrichment with deconvolution approaches, which present a compendium of newly generated gene signatures for 64 cell types, spanning multiple adaptive and innate immunity cells, hematopoietic progenitors, epithelial cells, and extracellular matrix cells derived from thousands of expression profiles [29–31]. Specifically, the ES of 64 candidate cell types were estimated by 489 gene set and the primary ES of all gene signatures corresponding to target cell were further averaged. Through this procedure, a cell matrix including 64 cell types and corresponding RCC samples numbers was generated and used to correlation analysis. Immune cells can be further classified into 9 categories, including CD4 T-cell subpopulations, CD8 T-cell subpopulations, gamma delta T cells (Tgd cells), monocyte/macrophage subpopulations, B-cell subpopulations, granulocyte subpopulations, nature killer (NK) cells, dendritic cell (DC) subpopulations, and NKT cells subpopulations. Finally, three summary score based on 64 immune and stromal cell types including microenvironment-score, immune-score, and stroma-score were also evaluated for further analyses [29–31].

### Identification of angiogenic subtypes using consensus clustering analysis

Consensus clustering analysis was performed for angiogenic subtypes discovery based on 183 angiogenesis associated genes in reference gene set. The consensus clustering method began by subsampling a proportion of genes features from the TPM matrix where each RCC sample was partitioned into up to K angiogenic groups. In addition, this subsampling process was repeated for 1000 times; these multiple clustering runs (CRs) were used to calculate consensus values (CVs) and to assess the stability of the identified angiogenic subtypes. Pairwise CVs, identified as the proportion of CRs in which two features (or items) were grouped together and stored in a matrix for each K angiogenic groups. Then, for each K angiogenic groups, a final consensus based hierarchical clustering (1-consensus values) was completed and pruned to K angiogenic groups. In this study, “ConsensusClusterPlus” package [32] in R software was used to identify TCGA KIRC angiogenic subtypes. After performing “*ConsensusClusterPlus ()*” function in R software, we obtained the cluster consensus (or item-consensus) results and was used to downstream analysis.

### Differential expression gene analysis and function annotation analysis

The RNA Sequencing data was primary quantified as raw read count expression matrix for differential expression gene (DEGs) analysis. In this study, the DEGs between two angiogenic groups (Cluster_1 versus cluster_2) were identified by edgeR packages in R software. A P value less than 0.05 and fold change value over 2.0 were considered as statistically significantly. To further investigate the potential mechanism of angiogenic pattern in TCGA KIRC patients, the DEGs between two angiogenic groups were used to enrichment function annotation analysis by using the ClusterProfiler and org.Hs.eg.db package in R software. In this section, an adjust P value < 0.05 was considered as statistically significantly.

### Copy number variations analysis and mutation analysis

GISTIC (Version 2.0.1) (https://gatkforums.broadinstitute.org) software was adopted to determine the copy number variations (CNVs) event enrichment of genomic regions including significantly amplified and deleted [33]. GISTIC method identifies genomic mutated regions that are overrepresented across all cancer samples, based on the amplitude of the mutations and their frequency, and a G-score was used to quantify the degree of overrepresentation. Then, a P value of each G-score was assigned, by comparing the G-score at each mutation region to a background G-score distribution, which was corrected using the false discovery rate (FDR) statistic, and resulting in multiple testing corrected q values. Finally, a cutoff value (q value = 0.25) was used to select candidate regions containing significantly overrepresented CNVs regions. Mutation annotation format (MAF) of somatic mutation data based on TCGA KIRC cohort was prepared for mutation analysis. The mutational loading between two angiogenic groups was compared by performing “maftools” package in R software [34].

### Differential methylation analysis and master regulator transcription factor analysis

By using the ChAMP package (Version 2.13.5) in R software, β value matrix was first filtered and normalized through the embedded *SWAN* method. The differential methylated probes (DMPs) between two angiogenic groups were identified by the “*champ.DMP ()*” function in ChAMP package with the parameters P value less than 0.05 and delta β value over 0.1 [35, 36]. To further study the relationship between methylation and tumor angiogenesis in RCC, we performed gene set enrichment analysis (GSEA) based on identified DMPs by using “*champ.GSEA ()*” function in ChAMP package. To identify enriched motifs and potential upstream master transcription factors (TFs), Homo Sapiens Comprehensive Model Collection (HOCOMOCO Version 11) [36] TFs binding models were used as input for Hypergeometric Optimization of Motif EnRichment (HOMER Version 4.10) software to find motif occurrences in SEs region. Fisher’s exact test and Benjamini–Hochberg multiple hypothesis testing were used in motif enrichment analysis to correct the background of all DMPs regions. The motif occurred over 10 times and 95% confidence interval (CI) of the Odds Ratio (OR) value greater than 1.1 was considered as significantly enriched. Finally, the get.TFs () function embedded in Enhancer Linking by Methylation/Expression Relationships (ELMER) package was used to identify the master regulator TFs. For each enriched motif, get.TFs () function takes the mean DNA methylation (MDM) of target sites that contain the motif occurrence and compares this MDM to the expression of human TF.

### Statistical analysis

Continuously variables presented as median and quartiles, or mean and standard deviation (SD), depending on the distribution pattern (normal or non-normal) of each variable which was tested by performing Shapiro–Wilk test in R software. On the other hand, categorical code variables were reported as factor frequencies and proportions. The statistical methods used to test the difference between two angiogenic groups included two independent samples t-test for mean values, Mann–Whitney U-test for median values, and Fisher’s exact test or Chi-square test for frequencies and proportions variables. Kruskal–Wallis test was performed to compare the difference between multiple groups. The correlation between two variables (continuously) was studied and tested by Pearson or Spearman coefficients based on the distribution pattern of variables. The prognostic value of ten independent angiogenic scores were evaluated by univariate Cox regression model (survival package), Kaplan–Meier curve (survival package), and time dependent receiver operating characteristic curve (ROC) (time ROC and timereg packages) in R software. On the other hand, the prognostic value of VEGF signal score, hypoxia score, ICI related score, angiogenesis-related genes, methylation site signal, and master transcription factors (TFs) were evaluated by Kaplan–Meier curve based on survival package in R software. In this study, all statistical tests were performed in R software (Version 3.5.1) and a two-tailed P value < 0.05 was considered as a statistically significant level.

## Result

### Prognosis-related angiogenic signals in TCGA KIRC cohort

**Fig. 1:**
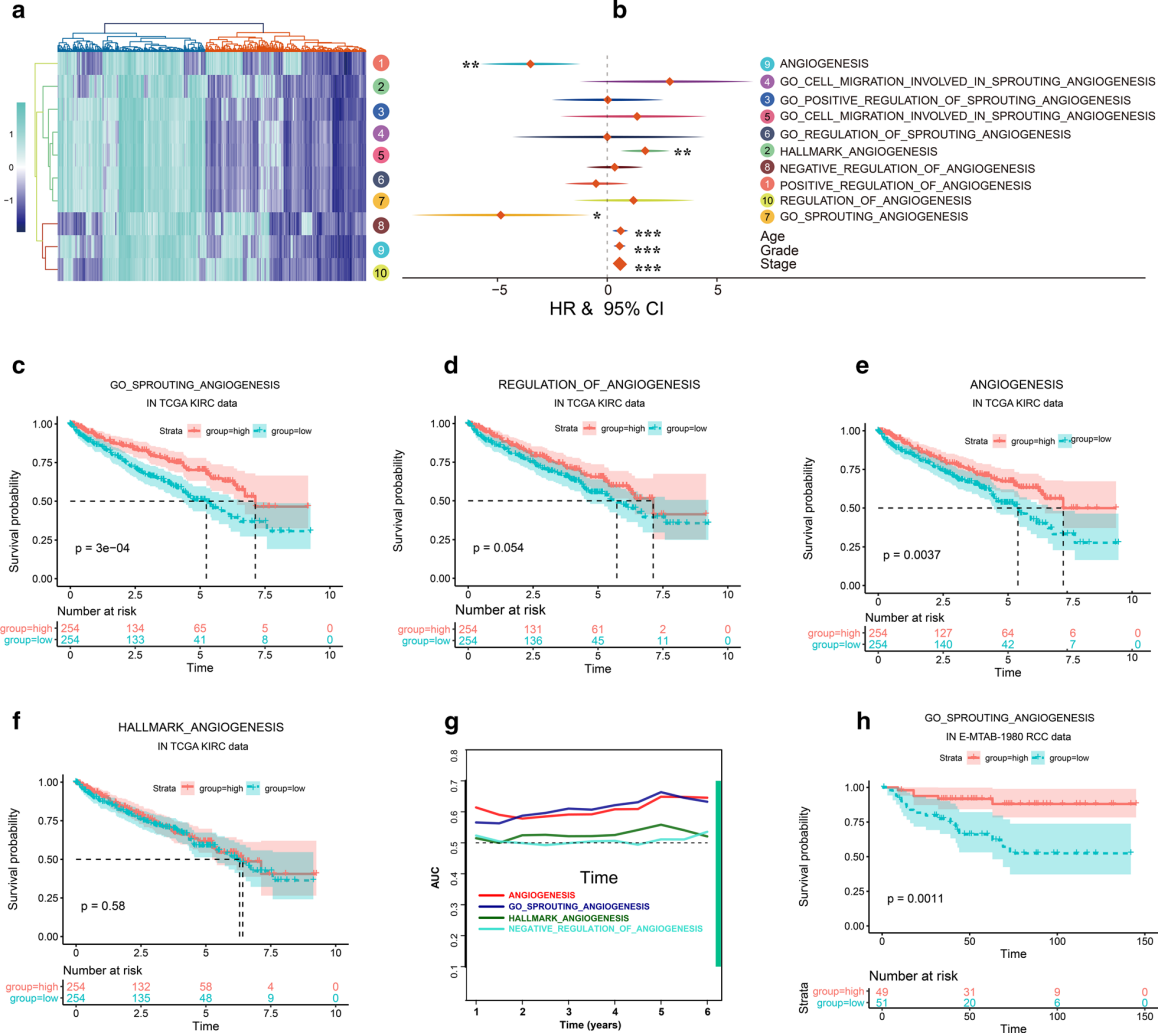
Prognosis-related angiogenesis signals in TCGA KIRC cohort. **a** Heat-map analysis showed 10 angiogenesis signaling associated GSVA scores in TCGA KIRC Cohort. Hierarchical clustering analysis based on 10 independent angiogenesis score showed that 539 TCGA KIRC samples were cluster into 2 angiogenesis subgroups; **b** univariate Cox regression analysis showed that No. 2, No. 7, No. 9 angiogenesis score were associated with the prognosis of TCGA KIRC patients and No. 7 angiogenesis score showed highest HR. Kaplan–Meier survival curve analysis showed the prognosis effect of No. 7 (**c**), No. 9 (**d**), No. 2 (**e**), No. 10 (**f**) in TCGA KIRC patients; **g** time dependent AUC analysis showed that the AUC value of No. 7 angiogenesis score was the highest during the observation period of 2–5 years in TCGA KIRC cohort. **h** Kaplan–Meier survival curve analysis showed the prognosis effect of No. 7 (**c**) in E-MTAB-1980 RCC patients

**Fig. 2:**
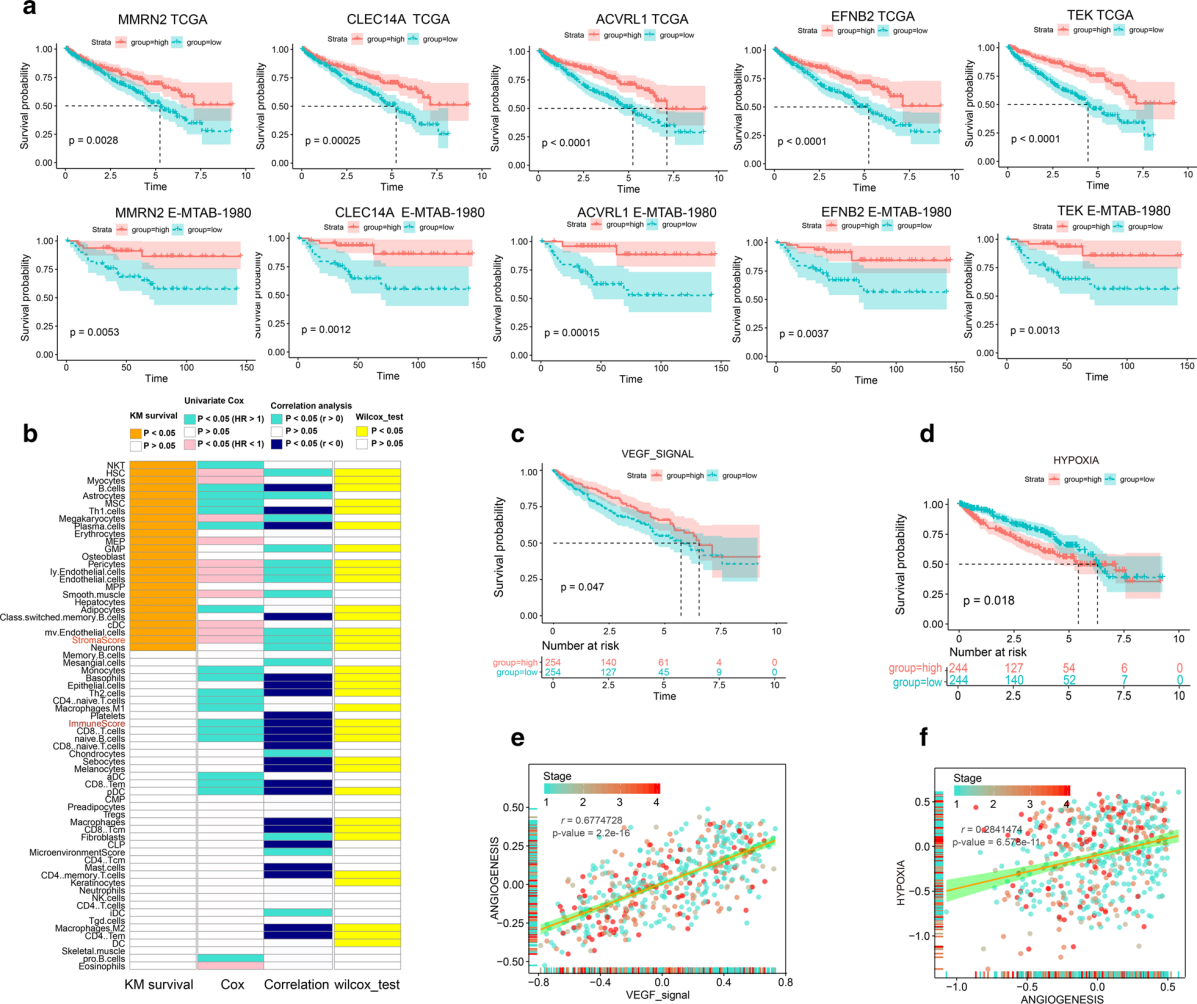
The relationship between tumor microenvironment associated signals and angiogenesis score in TCGA KIRC patients. MMRN2, CLEC14A, ACVRL1, EFNB2, and TEK in No. 7 candidate angiogenesis gene set were associated with overall survival in both TCGA-KIRC and E-MTAB-1980 KIRC data **a**, **b** prognosis associated immune cells were showed on the left side of figure; correlation between angiogenesis and immune cells was showed on the right side of figure; Kaplan–Meier survival curve analysis showed the prognosis effect of VEGF signal (**c**) and hypoxia (**d**) signal in TCGA KIRC patients; **e** correlation between angiogenesis and VEGF signal in TCGA KIRC samples; **f** correlation between angiogenesis and hypoxia signal in TCGA KIRC samples

First, we numbered 10 independent angiogenesis reference gene sets for subsequent analysis (Additional file 4: Table S1). Then, gene set variation analysis was performed to calculate the angiogenic score each TCGA KIRC sample. Hierarchical clustering analysis based on 10 independent angiogenic score showed that 539 TCGA KIRC samples were cluster into 2 angiogenesis subgroups (Fig. 1a). Because the distribution patterns of the angiogenesis score in RCC samples were not consistent (Fig. 1a), we analyzed the prognosis effects of 10 independent angiogenesis score in TCGA KIRC cohort respectively. Univariate Cox regression analysis showed that No. 2, No. 7, No. 9 angiogenesis score were associated with the prognosis of TCGA KIRC patients and No. 7 angiogenesis score showed highest hazard ratio (HR) (Fig. 1b). On the other hand, Kaplan–Meier survival curve analysis showed that No. 7, No. 9 angiogenesis score were positively correlated with the survival of TCGA KIRC patients (Fig. 1c–f) (P value = 0.0000 and P value = 0.0037, respectively). In addition, during the observation period of 2–5 years in TCGA KIRC cohort, the AUC value of No. 7 angiogenesis score was the highest (Fig. 1g). Taken together, No. 7 angiogenesis associated gene set was selected as reference gene set (and angiogenesis score) for subsequent analysis. Consistent with TCGA results, the angiogenesis score in E-MTAB-1980 data were positively correlated with the survival of RCC patients (Fig. 1h) (P value = 0.0011). In TCGA-KIRC cohort, higher clinicopathological patients showed lower angiogenesis score (Additional file 2: Figure S1A-B). Next, correlation analysis was performed to identify the feature gene of angiogenesis score. MMRN2 (multimerin-2), CLEC14A (C-type lectin 14A), ACVRL1 (Activin A Receptor Like Type 1), EFNB2 (Ephrin B2) and TEK (TEK receptor tyrosine kinase) in No.7 candidate angiogenesis gene set showed highest correlation coefficient with angiogenesis score in TCGA-KIRC patients (Additional file 5: Table S2). In addition, all of them were associated with overall survival in both TCGA-KIRC and E-MTAB-1980 KIRC data (Fig. 2a).

### Relationship between angiogenesis signal and tumor microenvironment in TCGA KIRC cohort

Tumor microenvironment is closely related to the aberrant angiogenesis and metastasis of cancer. In this section, we systematically analyzed the relationship between tumor microenvironment associated signals and angiogenesis score in TCGA KIRC patients. First, we analyzed the prognosis effect of each immune cells in TCGA KIRC patients. Specifically, Univariate Cox analysis of immune cells was performed, we found NKT, B cells, Th1 cells, Th2 cells, M1 macrophages, CD8 T cells, aDC, CD8 Tem, pDC, pro B cells, and summary immune score were unfavorable prognosis, cDC, eosinophils, eosinophils, and summary stroma score were favorable prognosis for TCGA KIRC patients (Fig. 2b). In addition, Kaplan–Meier analysis of 64 cell types was also conducted, NKT, B cells, plasma.cells, Th1 cells, class switched memory B cells, cDC, and stroma score were prognostic (P value < 0.05) (Fig. 2b). We then performed correlation analysis and found that angiogenesis score in TCGA KIRC patients was significantly positively related to the infiltration intensity of iDC, stroma score, and microenvironment score (Fig. 2b). On the other hand, B cells, Th1 cells, class switched memory B cells, Th2 cells, CD8 naïve T cells, naïve B cells, CD8 T cells, CD8 Tcm, macrophages, CD4 Tem, M2 macrophages and summary immune score were significantly negatively related to the angiogenesis score in TCGA KIRC patients (Fig. 2b). Next, we studied the hypoxia signal, VEGF signal, and glycolysis signal in TCGA KIRC samples. Overall survival (OS) analysis results showed that VEGF signal score was favorable prognosis (P value = 0.047) (Fig. 2c) and hypoxia signal score was unfavorable prognosis (P value = 0.018) (Fig. 2d) for TCGA KIRC patients. Interesting, both VEGF signal score and hypoxia signal score were significantly positively related to the angiogenesis score in TCGA KIRC patients (Fig. 2e, f).

### Angiogenesis subtypes of TCGA KIRC patients

**Fig. 3:**
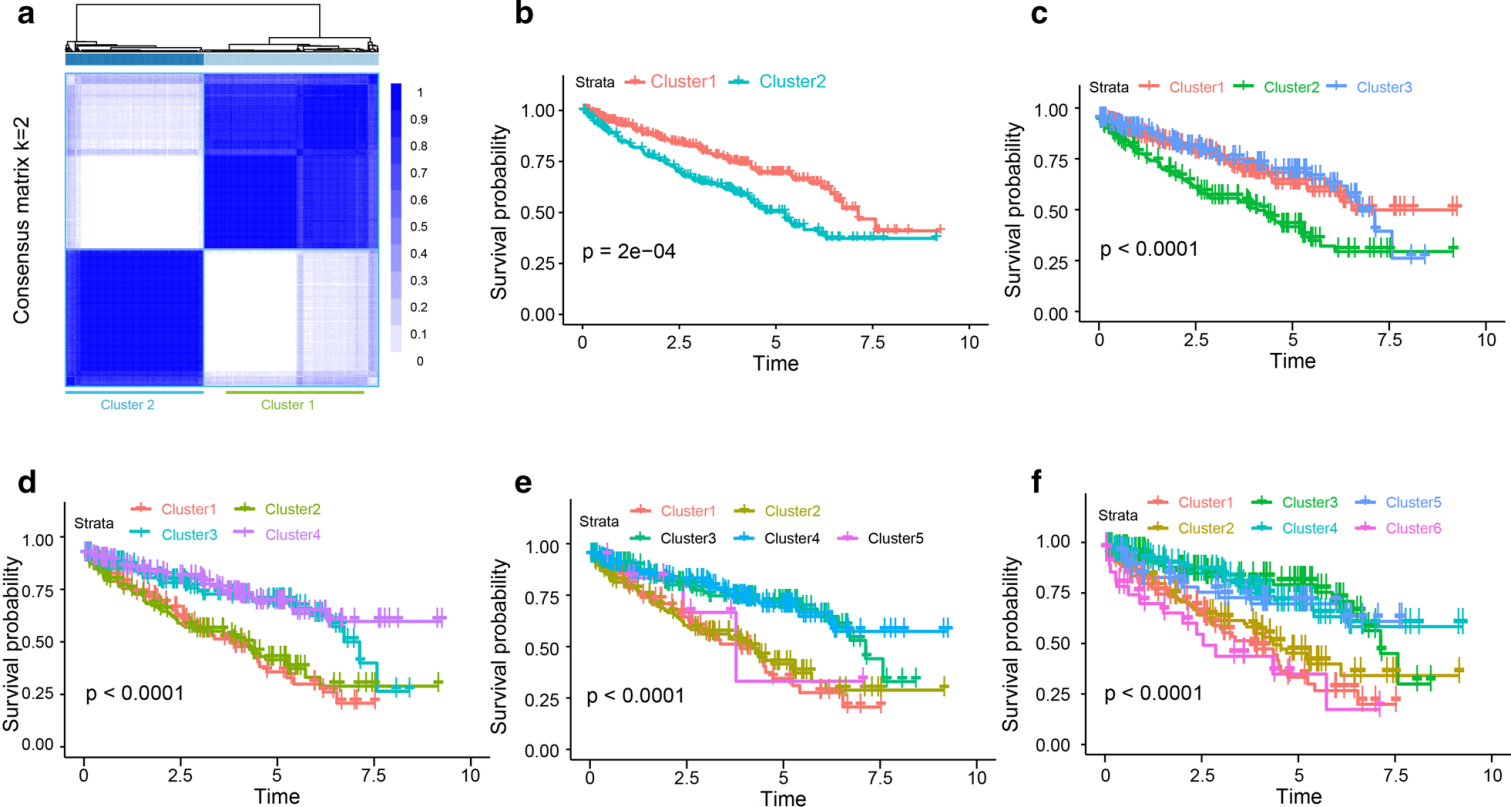
Angiogenesis subtypes of TCGA KIRC patients. **a** Unsupervised consensus clustering analysis identified two angiogenesis subtypes, namely Cluster_1 and Cluster_2. Clearly, K = 2 seemed to be a relatively stable distinction of the samples in the TCGA KIRC dataset; **b** patients belonging to Cluster_1 showed better prognosis in TCGA KIRC cohort. OS analysis based on 3–6 clusters (**c**, **d**, **e**, **f**) of KIRC patients were also conducted. These cluster are inseparable in prognosis

**Fig. 4:**
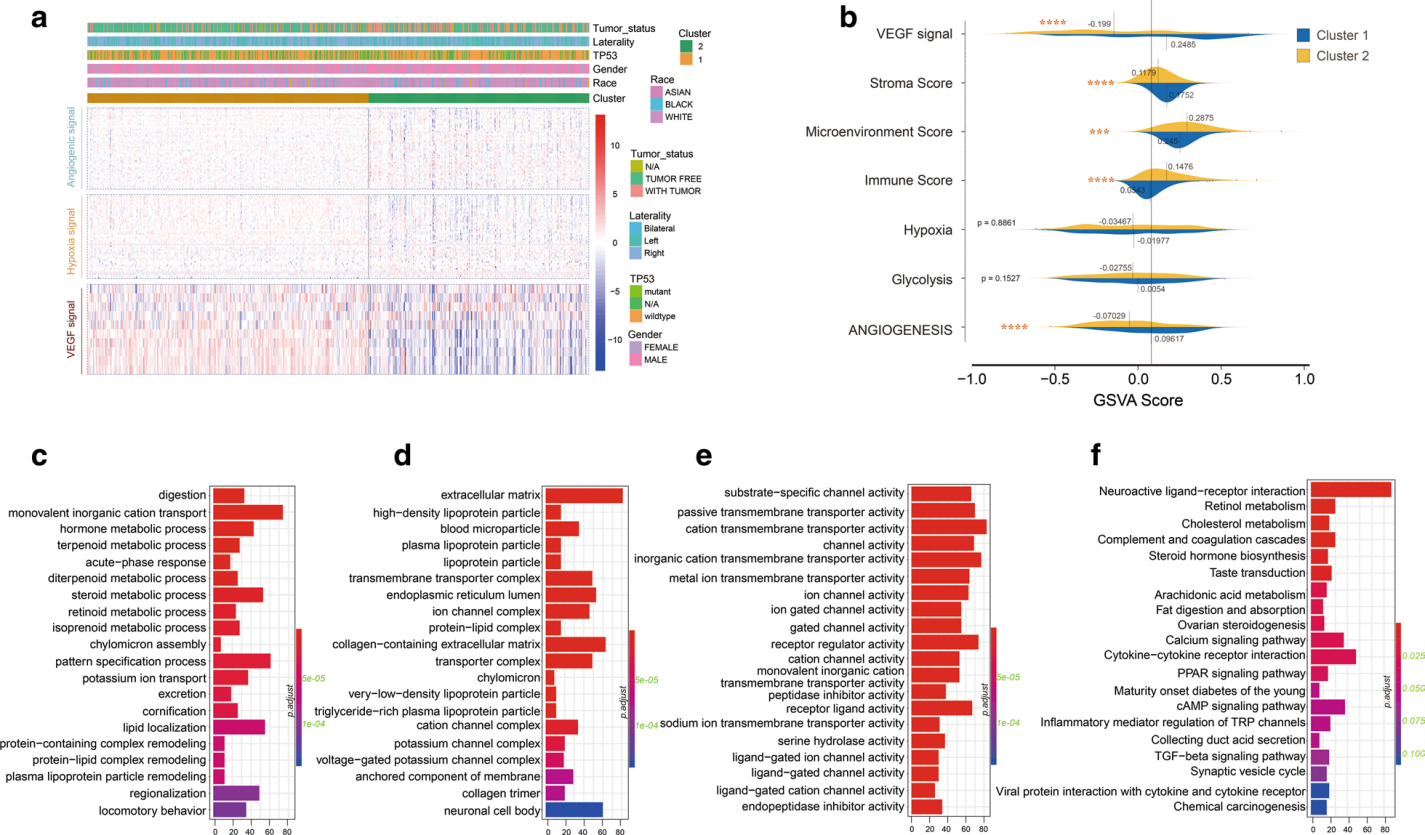
Clinical and molecular characteristics between two angiogenesis subgroups. **a** Heat-map analysis showed KIRC patients in Cluster_2 showed higher VEGF signal, hypoxia signal, and angiogenesis score; **b** Compared with Cluster_2, RCC patients in Cluster_1 showed higher VEGF signal, stroma score, immune score, microenvironment score, and angiogenesis score; **c**–**d** DEGs between Cluster_1 and Cluster_2, and found these genes were significantly enriched in metabolism-related pathways; * represent P value less than 0.05

Based on the 183 angiogenic related gene expression profiles in No. 7 gene set of 539 patients with RCC in the TCGA KIRC dataset, unsupervised consensus clustering analysis identified two angiogenesis subtypes, namely Cluster_1 and Cluster_2 (Fig. 3a). Clearly, K = 2 seemed to be a relatively stable distinction of the samples in the TCGA KIRC dataset with clustering stability increasing from K = 2 ~ 6 (Additional file 2: Supplementary Figure S1C-F). In addition, the survival of RCC patients in Cluster_2 subtype was obviously shorter than Cluster_1 subtype (P < 0.0000) (Fig. 3b). On the other hand, OS analysis based on 3 ~ 6 clusters (K = 3 ~ 6) of KIRC patients were also conducted, but these cluster are inseparable in prognosis (Fig. 3c–f), which was meaningless in clinical, therefore we finally selected 2 classification (K = 2) for further study. Clinical and molecular characteristics including TP53 mutation, gender, tumor laterality, and race weren’t different between the two angiogenesis subgroups (Fig. 4a). Compared with Cluster_2, RCC patients in Cluster_1 showed higher VEGF signal, stroma score, immune score, microenvironment score, and angiogenesis score (Fig. 4a, b). To obtain deeper insights into the function of angiogenesis signal in KIRC, we performed function annotation analysis based on DEGs between Cluster_1 and Cluster_2 (Additional file 6: Table S3, Additional file 3: Supplementary Figure S2A), and found these genes were significantly enriched in metabolism-related pathways (Fig. 4c–f).

### CNVs and somatic mutation events in two angiogenesis subgroups of TCGA KIRC patients

**Fig. 5:**
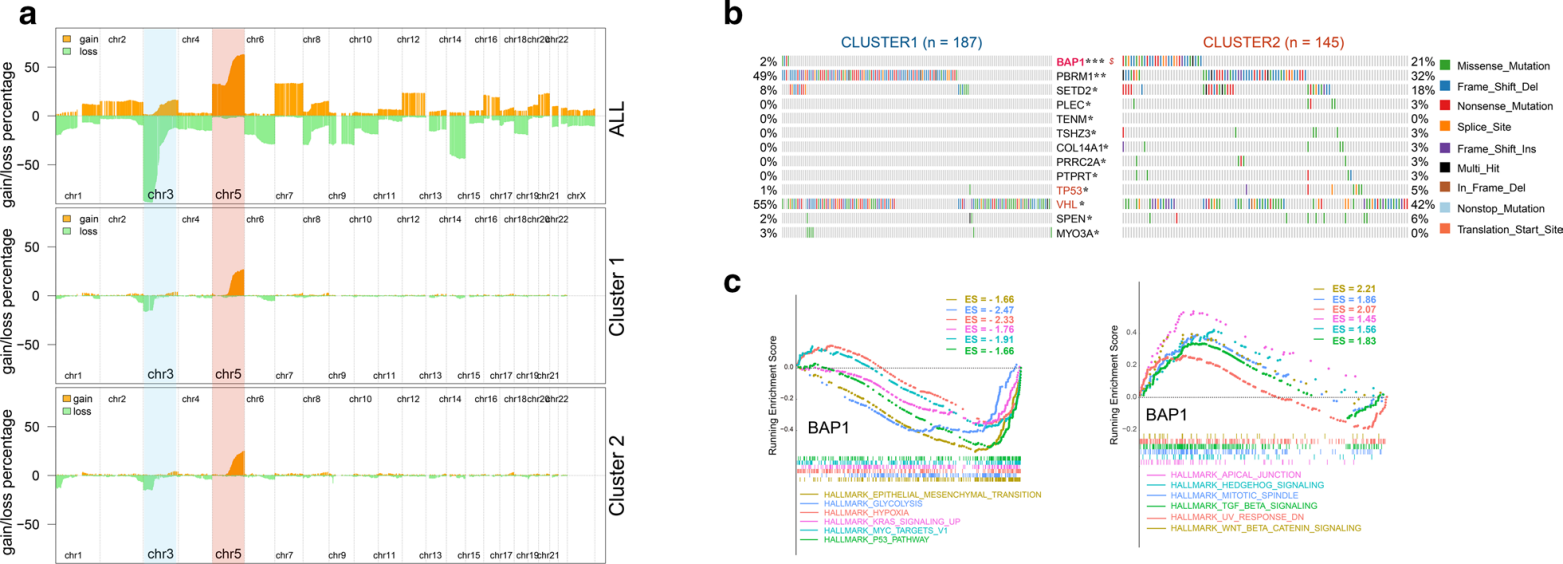
Copy number variations and somatic mutations between two angiogenesis subgroups. **a** A deletion of Chr 3 accompanied with an amplification of Chr 5 was enriched in all TCGA KIRC cases (upper panel). However these peaks in Chr 3 and Chr 5 did not differ significantly between the Cluster_1 and Cluster_2 angiogenesis subgroups (middle and lower panel); **b** only BAP1 showed significant mutation differences between two angiogenesis subgroups of TCGA KIRC patients; **c** GSEA showed that BAP1 in TCGA KIRC samples did not directly enriched in angiogenesis pathway, but significantly enriched in TP53, MYC target, hypoxia, epithelial mesenchymal transition (EMT), glycolysis, TGF-β signal related pathways; * represent P value less than 0.05; $ represent adjust P value less than 0.05

To explore the molecular mechanisms of angiogenesis variation in KIRC, CNVs and somatic mutations from TCGA database were analyzed. As shown in Fig. 5a, a deletion of Chr 3 accompanied with an amplification of Chr 5 was enriched in all TCGA KIRC cases (n = 530). However these peaks in Chr 3 and Chr 5 did not differ significantly between the Cluster_1 (n = 296) and Cluster_2 (n = 234) angiogenesis subgroups. In addition, other chromosomal positions did not show obvious peak changes in Cluster_1 and Cluster_2 angiogenesis subgroups (Fig. 5a). These results indicated that large-scale chromosomal variation (CNV) may not be the main cause of the differences of angiogenesis in KIRC patients. Then somatic mutations were investigated between the cases in Cluster_1 and Cluster_2 angiogenesis subgroups. The frequency of somatic mutations between cases with high angiogenesis signal in Cluster_1 and low angiogenesis signal in Cluster_1 was no statistical significance after correction (87.7% vs 87.59% mutations) (Additional file 3: Supplementary Figure S2B-C). High frequency genes including Von Hippel–Lindau Tumor Suppressor (VHL), Polybromo 1 (PBRM1), Mucin 16, Cell Surface Associated (MUC16), Mechanistic Target of Rapamycin Kinase (MTOR), and SET Domain Containing 2, Histone Lysine Methyltransferase (SETD2) were detected in both KIRC cases in Cluster_1 and Cluster_2 angiogenesis subgroups (Additional file 3: Supplementary Figure S2B-C). Through statistical analysis, we found that only BAP1 showed significant mutation differences between two angiogenesis subgroups of TCGA KIRC patients (Fig. 5b). Gene Set Enrichment Analysis (GSEA) showed that BAP1 in TCGA KIRC samples did not directly enriched in angiogenesis pathway, but significantly enriched in TP53, MYC target, hypoxia, epithelial mesenchymal transition (EMT), glycolysis, TGF-β signal related pathways (Fig. 5c).

### Differential methylation sites in two angiogenesis subgroups of TCGA KIRC patients

**Fig. 6:**
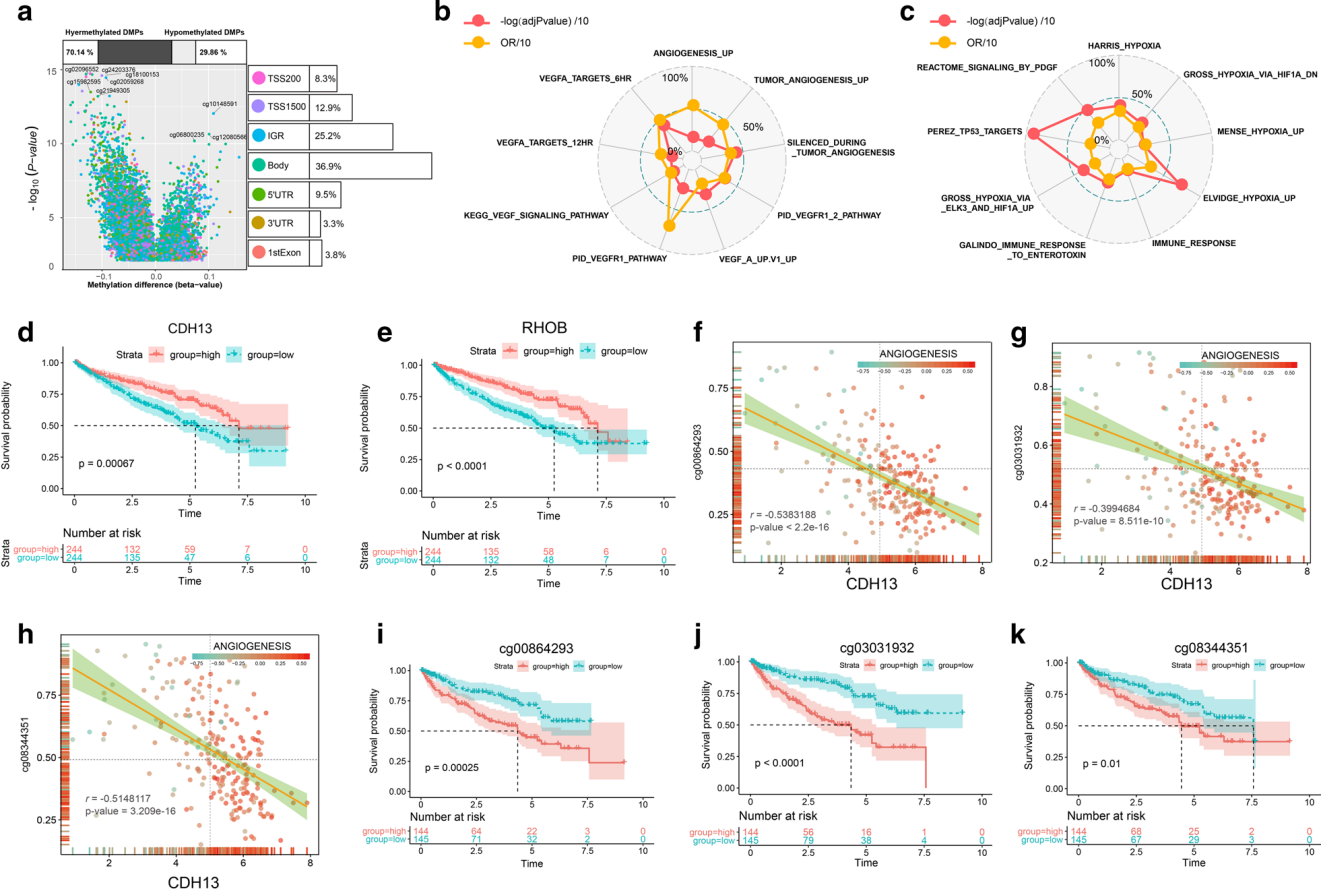
Differential methylation sites in two angiogenesis subgroups of TCGA KIRC patients. **a** Most differential methylation sites (70.14%) between Cluster_1 and Cluster_2 angiogenesis subgroups were presented as hypermethylation sites. Gene body and intergenic region shared the highest proportion of differential methylation sites; **b**, **c** GSEA analysis showed that those differential methylation sites were significantly enriched in tumor angiogenesis, VEGFA, immune, TP53 targets, and hypoxia-associated pathways; **d**, **e** higher CDH13 and RHOB expression in KIRC patients were associated with better OS according to *Kaplan–Meier* survival log-rank test; **f**, **g**, **h** Methylation levels of CDH13 related enhancer including cg00864293, cg03031932, and cg08344351 were negatively correlated with the expression level of CDH13; **i**, **j**, **k** Lower methylation levels of CDH13 related enhancer including cg00864293, cg03031932, and cg08344351 showed better OS

In this section, we identified 59,654 differential methylation sites (P value < 0.05 and |delta β|> 0.1) in the samples of 187 Cluster_1 and 145 Cluster_2 RCC samples (Fig. 6a). Most differential methylation sites (70.14%) between Cluster_1 and Cluster_2 angiogenesis subgroups were presented as hypermethylation sites (Fig. 6a). Gene body and intergenic region shared the highest proportion of differential methylation sites, which accounting for 36.9% and 25.2%, respectively (Fig. 6a). Then we performed GSEA analysis to study the biological functions of differential methylation sites in KIRC patients and found that those differential methylation sites were significantly enriched in tumor angiogenesis, vascular endothelial growth factor A (VEGFA), immune, TP53 targets, and hypoxia-associated pathways (Fig. 6b, c). By performing ELMER analysis, we found 9 angiogenesis related genes in No. 7 reference gene set were significantly correlated with the methylation level of corresponding enhancer (Table 1). In addition, one angiogenesis related genes was regulated by the methylation of multiple enhancer sites in KIRC samples. For example, the expression of Cadherin 13 (CDH13) and Ras Homolog Family Member B (RHOB) in KIRC were regulated by 4 independent enhancers respectively (Fig. 6f–h, Table 1). Higher CDH13 and RHOB expression in KIRC patients were associated with better OS (P value = 0.00067 and P value = 0.0000) (Fig. 6d, e) according to *Kaplan–Meier* survival log-rank test. In addition, lower methylation levels of CDH13 related enhancer including cg00864293, cg03031932, and cg08344351 showed better OS (P value = 0.00025, P value = 0.0000, and P value = 0.01) (Fig. 6f–k).

**Table 1:** Angiogenesis related genes in No. 7 reference gene set were significantly correlated with the methylation level of corresponding enhancer

Symbol	Probe	Distance	Raw.p	Pe
ANG	cg24162781	63,309	4.1017E-19	0.000999001
CDH13	cg08344351	1,153,078	7.57956E-13	0.000999001
CDH13	cg00864293	1,073,606	8.19426E-14	0.000999001
CDH13	cg03031932	1,113,268	5.77968E-09	0.000999001
CDH13	cg16583186	1,134,045	4.70367E-10	0.000999001
COL4A3	cg18018313	372,087	1.44211E-16	0.000999001
COL4A3	cg12302987	2,752,319	1.52068E-16	0.000999001
HTATIP2	cg09118558	252,588	1.75087E-11	0.000999001
PLG	cg22008851	22,694	1.39688E-09	0.000999001
RHOB	cg11426800	− 3,060,799	1.49831E-09	0.000999001
RHOB	cg01227744	2462	1.11331E-10	0.000999001
RHOB	cg02153681	− 65,617	7.00899E-10	0.000999001
RHOB	cg07493834	− 1,465,071	7.00899E-10	0.000999001
SPHK1	cg10616974	− 127,777	2.17239E-11	0.000999001
SPINK5	cg02154084	1,220,880	1.65585E-08	0.000999001
VEGFA	cg23906612	− 204,454	3.21951E-14	0.000999001
VEGFA	cg26464586	− 167,487	6.50071E-13	0.000999001

### Master transcription factors in regulating angiogenesis signal of TCGA KIRC patients

**Fig. 7:**
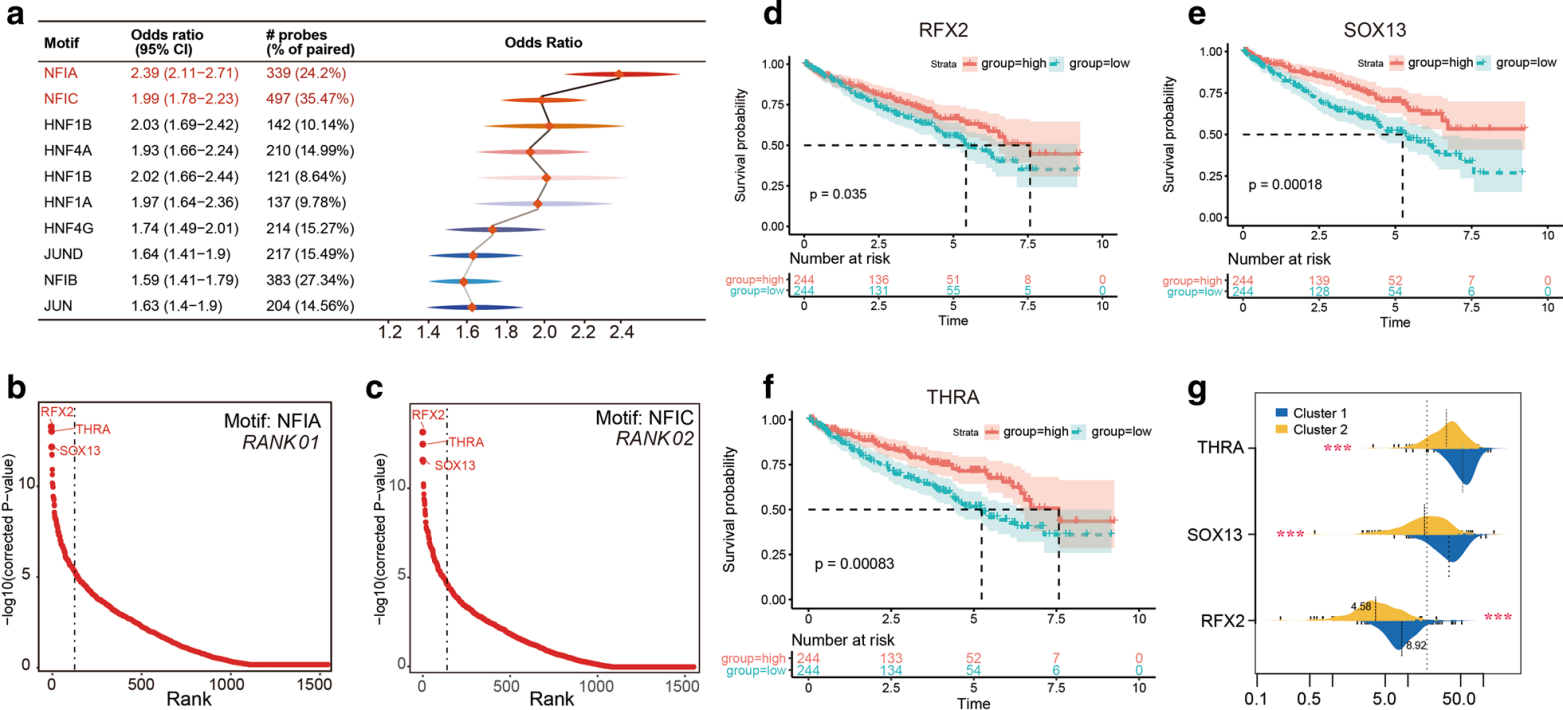
Master transcription factors in regulating angiogenesis signal of TCGA KIRC patients. **a** Motif enrichment analysis showed that NFIA and NFIC shared the highest odds ratio value and percentage; **b**, **c** transcription factors including RFX2, SOX13, and THRA were identified as TOP3 master TFs in both NFIA and NFIC motif; Higher RFX2 (**d**), SOX13 (**e**), and THRA (**f**) expression in KIRC patients were associated with better OS according to Kaplan–Meier survival log-rank test; **g** compared with Cluter_2, RCC patients in Cluster_1 showed higher RFX2, SOX13, and THRA expression. * represent P value less than 0.05

The small sequences within largely DNA elements which can be used to control the transcription of target genes in mammalian cells care called regulatory motif. In this section, we totally identified 191 significantly enriched motif in the differential methylation sites between Cluster_1 and Cluster_2 angiogenesis subgroups base on motif enrichment analysis. Of these motif, Nuclear Factor I A (NFIA) and Nuclear Factor I C (NFIC) shared the highest odds ratio (OR) value and percentage (Fig. 7a, Additional file 7: Table S4). By enriching the motif signatures of differential methylation sites, we can identify candidate master TFs that bind to methylation regions affected by the absence or accumulation of DNA methylation. TFs including Regulatory Factor X2 (RFX2), SRY-Box Transcription Factor 13 (SOX13), and Thyroid Hormone Receptor Alpha (THRA) were identified as TOP3 master TFs in both NFIA and NFIC motif (Fig. 7b, c, Additional file 8: Table S5). In addition, higher RFX2, SOX13, and THRA expression in KIRC patients were associated with better OS (P value = 0.035, P value = 0.00018, and P value = 0.00083) (Fig. 7d, f) according to *Kaplan–Meier* survival log-rank test. Finally, compared with Cluter_2, RCC patients in Cluster_1 showed higher RFX2, SOX13, and THRA expression (Fig. 7g).

## Discussion

Angiogenesis is a hallmark event in cancer patients [37]. In normal tissues, angiogenesis is a complex multi-step process associated with changes in cellular adhesive interactions between adjacent pericytes, equilibrium cells, soluble factors, and surrounding extracellular matrix components [38]. During the process of formatting capillary buds, activated equilibrium cells reorganize their cytoskeleton and express cell adhesion molecules including proteolytic enzymes, integrins family proteins, selectins family proteins, and remodel their adjacent extracellular matrix components [38]. Pro-angiogenic factors including autocrine and paracrine must be present to induce equilibrium cell proliferation, elongation, orientation, migration, and differentiation leading to the re-establishment and formation of intact micro-vessels. During the process of tumorigenesis, malignant cells express high level of pro-angiogenic growth factor to promote the formation of new blood vessels [6]. Specifically, aberrant angiogenesis in tumorigenesis and metastasis is closely related to tumor microenvironment [39, 40].

In this study, we first systematically analyzed the relationship between the tumor immune microenvironment and angiogenesis signal in TCGA KIRC samples. The stroma and microenvironment scores in TCGA KIRC patients was positively related to angiogenesis score. It is relevant to know that blood vessel is the basic compartment of tissue stroma. Besides, we found the infiltration intensity of iDC was positively related to the angiogenesis score of TCGA KIRC patients. Previous study showed that DC can inhibit or stimulate angiogenesis mainly depending on their status and subset specificity. Specifically, DC stimulate angiogenesis by secreting cytokines, promoting the proangiogenic activity of T cells, and trans-differentiating into the endothelial cells of new micro-vessels [41]. In an in vitro study, Suen et al. reported that iDC promotes angiogenesis in the early stage of endometriosis by secreting anti-inflammatory cytokine IL-10 [42]. In addition, in an in vivo study, Fainaru et al. found depletion of DCs in a transgenic animal model that allows for their conditional ablation completely abrogated basic fibroblast growth factor-induced angiogenesis, and significantly inhibited tumor growth in these mice [43]. Therefore, we speculate that promote DC maturation may act by not only augmenting the host immune response to the cancer but also by suppressing cancer angiogenesis. Tumor associated macrophage, an important compartment of tumor stroma, was showed to promote cancer cell proliferation and stimulate angiogenesis [37]. Similarly, our analysis results showed that M2 macrophages were related to the angiogenesis score of TCGA KIRC patients. In this study, both VEGF signal score and hypoxia signal score were significantly positively related to the angiogenesis score in TCGA KIRC patients. Previous studied have showed that hypoxia and related HIF family proteins such as HIF-α proteins and HIF-β proteins can promote angiogenesis process in cancer patients [11, 12]. These results were further confirmation that angiogenesis score can effectively represent the angiogenesis and tumor microenvironment in TCGA KIRC cohort.

In our present study, correlation analysis found that MRN2, CLEC14A, ACVRL1, EFNB2, and TEK in No.7 candidate angiogenesis gene set showed highest correlation coefficient with angiogenesis score in TCGA-KIRC patients, and all of them were associated with overall survival in both TCGA-KIRC and E-MTAB-1980 KIRC data. MMRN2 is an extracellular matrix glycoprotein, it interfered with VEGF-VEGFR binding and led to anti-angiogenic effects [45–47]. CLEC14A is expressed on the vasculature of tumours, more than half of renal cell carcinomas were high expression [48]. CLEC14A is a tumour endothelial marker, it induces sprouting angiogenesis by directly binds to with MMRN2 [49].

To further explore the molecular mechanisms of angiogenesis variation in KIRC patients, we performed somatic CNV analysis and found chromosomal positions did not show obvious peak changes in Cluster_1 and Cluster_2 angiogenesis subgroups. Similarly, the frequency of somatic mutations between cases with high angiogenesis signal in Cluster_1 and low angiogenesis signal in Cluster_1 did not showed obvious changes. These result indicated that somatic CNV and mutations may not be the main cause of the differences of angiogenesis in KIRC patients. Through statistical analysis, we found that only BAP1 showed significant mutation differences between two angiogenesis subgroups of TCGA KIRC patients and GSEA results did not showed BAP1 gene directly enriched in angiogenesis pathway. BAP1 protein belongs to the ubiquitin C-terminal hydrolase subfamily of deubiquitinating enzymes and considered as a key regulator of many cancer-related pathways [50]. Germline alterations in BAP1 have been characterized as predisposing variants to familial melanocytic skin tumors [50]. In kidney cancer, BAP1 is an oncogene, provide an update on the function of the gene products, and may be exploited therapeutically [51]. Other studies have found that BAP1 protein is essential for kidney function and cooperates with VHL in KIRC [52, 53]. However, the regulatory mechanism of BAP1 on renal cancer angiogenesis remains to be further studied.

In this study, we found 9 angiogenesis related genes in No. 7 reference gene set were significantly correlated with the methylation level of corresponding enhancer regions. In addition, one angiogenesis related genes was regulated by the methylation of multiple enhancer sites in KIRC samples. For example, the expression of CDH13 and RHOB in KIRC were regulated by the methylation level of 4 independent enhancers regions respectively. CDH13, also known as T cadherin, is frequently silenced in many different cancers and considered as a tumor suppressor genes [54, 55]. In an in vitro study, Wang et al. reported that the methylation of CDH13 promoter region restores the angiogenesis, invasive capabilities, and migratory of cancer [56]. In addition, treatment of cancer cell lines with a histone deacetylase inhibitor or a de-methylating agent reactivated CDH13 expression resulted in angiogenesis and tumor metastasis. Overexpression of CDH13 in endothelial cells stimulates their migration and stimulates angiogenesis under pathological conditions, apparently by potentiating factors such as VEGF [57]. On the other hand, in an in vivo study, Hebbard et al. found CDH13 supports tumor angiogenesis and adiponectin association with the vasculature in a mice cancer model [58]. However, the regulatory mechanism of CDH13 on renal cancer angiogenesis remains to be further studied. RhoB protein, a member of Ras Homolog family (or Rho GTPase family) responsible for cell-cycle progression, adhesion, protein trafficking, and actin cytoskeleton-mediated motion, serves an important role in the intracellular signaling pathways, including the epidermal growth factor receptor (EGFR), PI3K/Akt/mTOR, and MYC pathways [59]. For example, RHOB protein can then inhibit or (in angiogenic states) enhance AKT Serine/Threonine Kinase activity, inhibit the EGFR receptor, facilitating MYC turnover, antagonize Ras/PI3K/mTOR signaling, and inhibit tumorigenesis [60]. In breast cancer, loss of RhoB had negatively (no) effect on cancer metastasis, RhoB overexpression result in decreased cancer metastasis to the liver, lungs, and lymph nodes of mouse. In kidney cancer, overexpression of RhoB significantly inhibits KIRC cell malignant phenotype, which indicated that RhoB play a cancer suppressive role in KIRC cells, raising its potential value in future therapeutic target for the patients of KIRC [61].

The distribution of differential methylation sites in the non-coding region of the genome including intergenic region and transcription start sites (TSS) indicated that the methylation of DNA regulatory elements could act as underlying mechanisms for oncogenic transcription in the cancer cell of KIRC patients. In this study, we identified 3 master TFs including SOX13, RFX2, and THRA play a potential role in regulating the angiogenesis in KIRC patients. In glioma cancer, SOX13 protein played an important role in the regulation of angiogenesis by the feedback loop of FUS/circ_002136/miR-138-5p/SOX13 axis [62].

Hypoxia-inducible factors (HIFs) are master regulators of angiogenesis in tumor microenvironments. HIF-1a promoter is normally repressed by methylation, but abnormal demethylation was reported in colon cancer [62]. HIF-1a expression was suppressed observed in a hematopoietic cell line, which is suppressed by a process dependent on HIF-1a promoter DNA methylation [63, 64]. Other genes’ DNA methylation can affect HIF-1a stability. VHL hypermethylation leads to HIF-1a activation and upregulation of HIF-1a target genes [65]. Twist plays an important role in tumor angiogenesis involved in metastasis, its promoter methylation is a common phenomenon in metastatic carcinomas [66]. HIC1 encodes a transcriptional repressor, and its targets genes are involved in angiogenesis. HIC1 promoter hypermethylation was found in many solid cancers [67]. In this study, we identified 5 genes including CLEC14A, which showed high correlation coefficient with the angiogenesis score of KIRC patients. Interestingly, CLEC14A promoter was highly methylated in LUAD, leading to downregulation of CLEC14A. CLEC14A acts as an antitumor role in LUAD. These evidences confirmed the association angiogenesis related genes expression profiles and epigenetic methylation.

However, there is still no literature report on the effects of RFX2 and THRA on tumor angiogenesis. Our study provides a potential target for future tumor angiogenesis and an alternative strategy for combined anti-angiogenic or target therapy. We would like to acknowledge that this study is not devoid of limitations. First, our present study has a drawback of retrospective design with selection bias, and it is also limited to a single center (TCGA) that provided all of the KIRC patient samples. Second, angiogenesis score will need further confirmation from other centers and larger prospective studies for their generalizability. Finally, all of the work in this study is purely correlative and does not get at any mechanistic explanation. The regulatory mechanism of mutated gene, epigenetic methylation, and master TFs on KIRC remains to be further studied.

## Conclusion

In this study, we developed a novel angiogenesis score and feature genes (MMRN2, CLEC14A, ACVRL1, EFNB2, and TEK) which was associated with tumor angiogenesis microenvironment and prognosis in KIRC patients. In addition, we identified two prognosis associated angiogenesis subtypes in TCGA KIRC cohort. Two angiogenesis subtypes in KIRC samples showed different angiogenesis score, immune score, stroma score, hypoxia score, VEGF signal score, and microenvironment score. DMA identified 59,654 differential methylation sites between two clusters and part of these sites were correlated with tumor angiogenesis genes including CDH13, COL4A3, and RHOB. In addition, RFX2, SOX13, and THRA were identified as top three master TFs in regulating angiogenesis signature in KIRC patients. In summary, our study indicate that evaluation the angiogenesis subtypes of KIRC based on angiogenesis signature with 183 genes and potential epigenetic mechanisms may help to develop more target treatments for KIRC patients.

## Supplementary Information

**Additional file 2: Figure S1.** The association between their scores and clinicopathological characteristics (A, B). Unsupervised consensus clustering analysis identified angiogenesis subtypes in TCGA KIRC cohort. When K = 3 (C), 4 (D), 5 (E), 6 (F), the boundary of angiogenesis subtypes was not clear

**Additional file 3: Figure S2.** The heatmap of differential genes and mutation events in two angiogenesis subgroups of TCGA KIRC patients. (A) The heatmap of 20 differential genes (including 10 up-regulated genes and 10 down-regulated ones) between Cluster_1 and Cluster_2 angiogenesis subgroups. (B,C) Mutation events in two angiogenesis subgroups of TCGA KIRC patients. The frequency of somatic mutations between cases with high angiogenesis signal in Cluster_1 and low angiogenesis signal in Cluster_1 did not showed obvious changes (87.7% vs 87.59% mutations). High frequency genes including VHL, PBRM1, Mucin 16, MUC16, MTOR, and SETD2 were detected in both KIRC cases in Cluster_1 and Cluster_2 angiogenesis subgroups.

**Additional file 4: Table S1.** Ten independent angiogenesis reference gene sets.

**Additional file 5: Table S2.** Ten independent angiogenesis reference gene sets.

**Additional file 6: Table S3.** Differential expression genes between Cluster_1 and Cluster_2

**Additional file 7: Table S4.** Significantly enriched motif in the differential methylation sites between Cluster 1 and Cluster 2 angiogenesis subgroups.

**Additional file 8: Table S5.** Master transcription factors in regulating angiogenesis signal of TCGA KIRC patients.

## Data Availability

The data generated are included in the manuscript and supplementary data.

## Abbreviations

KIRC: Kidney renal clear cell carcinoma
MSigDB: Molecular signatures database
GSVA: Gene set variation analysis
TCGA: The Cancer Genome Atlas
DMA: Differential methylation analysis
MTF: Master transcription factors
VEGF: Vascular endothelial growth factor
RCC: Renal cell carcinoma
HIF: Hypoxia inducible factor
TAMs: Tumor-associated macrophages
TADCs: Tumor-associated dendritic cells
TKI: Tyrosine kinase inhibitors
VEGFRs: Vascular endothelial growth factor receptors
FDA: America food and drug administration
mRCC: Metastatic renal cell carcinoma
MHC-I: Major histocompatibility complex class I
NCCN: National Comprehensive Cancer Network
RN: Radical nephrectomy
NSS: Nephron-sparing surgery
TR: Tumor reduction
HTSeq: High-throughput sequencing
TPM: Transcripts per kilobase million
ICI: Immune cell infiltration
SMs: Somatic mutations
CNVs: Copy number variations
NK: Nature killer cells
DC: Dendritic cell
CRs: Clustering runs
CVs: Consensus values
DEGs: Differential expression gene
FDR: False discovery rate
MAF: Mutation annotation format
DMPs: Differential methylated probes
GSEA: Gene set enrichment analysis
TFs: Transcription factors
HOCOMOCO: Homo Sapiens Comprehensive Model Collection
HOMER: Hypergeometric Optimization of Motif EnRichment
CI: Confidence interval
OR: Odds ratio
ELMER: Enhancer Linking by Methylation/Expression Relationships
SD: Standard deviation
OS: Overall survival
CNV: Chromosomal variation
VHL: Von Hippel–Lindau Tumor Suppressor
PBRM1: Polybromo 1
MUC16: Mucin 16
MTOR: Mechanistic Target of Rapamycin Kinase
SETD2: SET Domain Containing 2
EMT: Epithelial mesenchymal transition
VEGFA: Vascular endothelial growth factor A
CDH13: Cadherin 13
RHOB: Ras Homolog Family Member B
NFIA: Nuclear Factor I A
NFIC: Nuclear Factor I C
RFX2: Regulatory Factor X2
SOX13: SRY-Box Transcription Factor 13
THRA: Thyroid Hormone Receptor Alpha
TSS: Transcription start sites MMRN2 multimerin-2
CLEC14A: C-type lectin 14A
ACVRL1: Activin A Receptor Like Type 1
EFNB2: Ephrin B2
TEK: TEK receptor tyrosine kinase
