# Supplementary figures and images for: Multi-omics analysis of tumor angiogenesis characteristics and potential epigenetic regulation mechanisms in renal clear cell carcinoma
Source: Cell Commun Signal. 2021 Mar 24;19:39. doi: 10.1186/s12964-021-00728-9 (PMC7992844; doi:10.1186/s12964-021-00728-9)

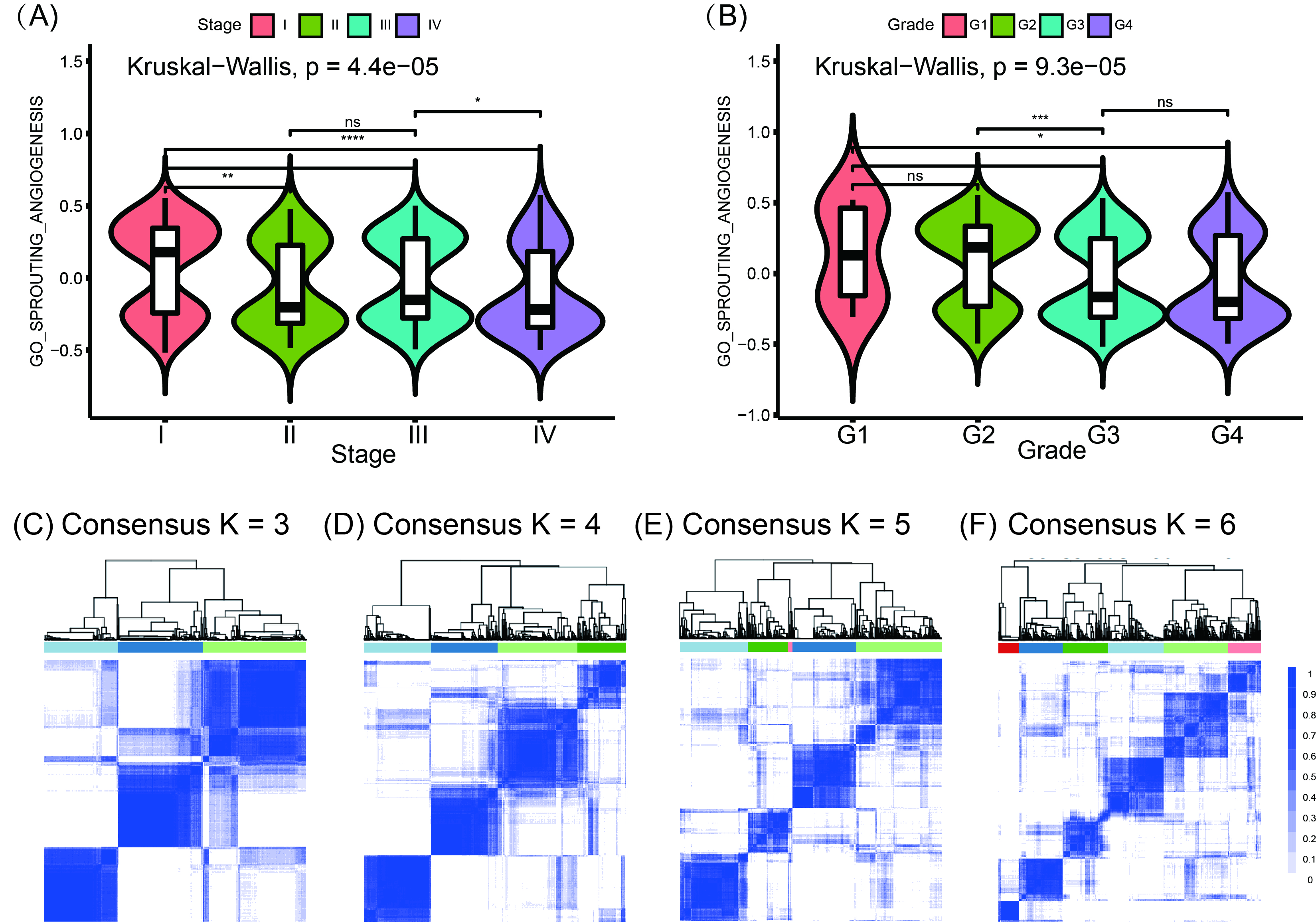

Supplement: Supplementary file 2 — Additional file 2: Figure S1. The association between their scores and clinicopathological characteristics (A, B). Unsupervised consensus clustering analysis identified angiogenesis subtypes in TCGA KIRC cohort. When K = 3 (C), 4 (D), 5 (E), 6 (F), the boundary of angiogenesis subtypes was not clear [file 12964_2021_728_MOESM2_ESM.jpg]

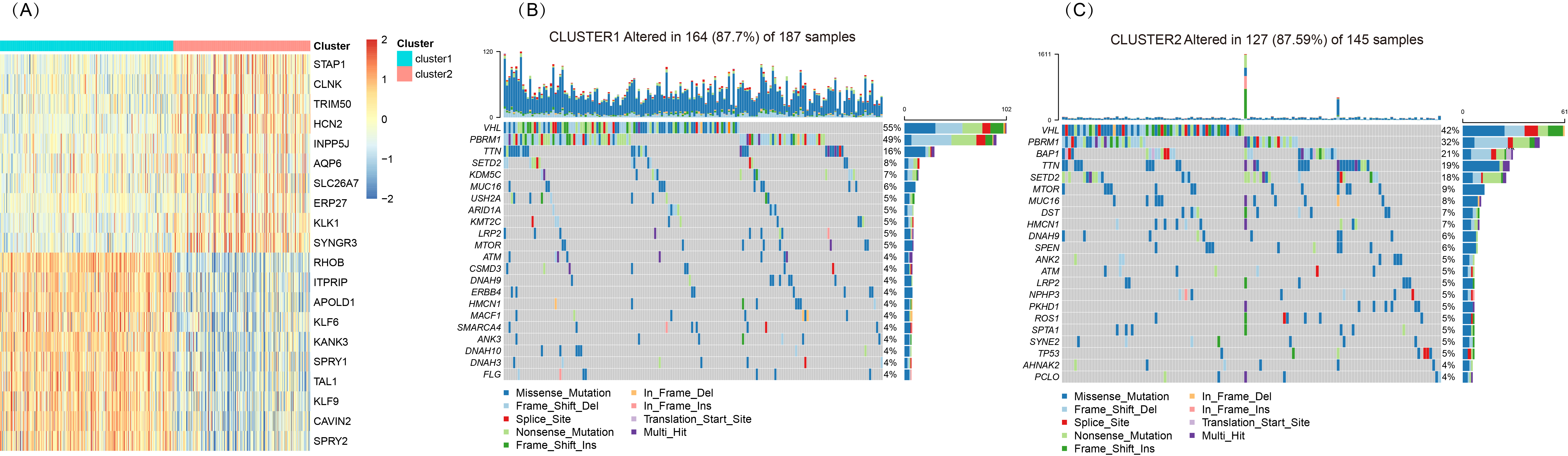

Supplement: Supplementary file 3 — Additional file 3: Figure S2. The heatmap of differential genes and mutation events in two angiogenesis subgroups of TCGA KIRC patients. (A) The heatmap of 20 differential genes (including 10 up-regulated genes and 10 down-regulated ones) between Cluster_1 and Cluster_2 angiogenesis subgroups. (B,C) Mutation events in two angiogenesis subgroups of TCGA KIRC patients. The frequency of somatic mutations between cases with high angiogenesis signal in Cluster_1 and low angiogenesis signal in Cluster_1 did not showed obvious changes (87.7% vs 87.59% mutations). High frequency genes including VHL, PBRM1, Mucin 16, MUC16, MTOR, and SETD2 were detected in both KIRC cases in Cluster_1 and Cluster_2 angiogenesis subgroups. [file 12964_2021_728_MOESM3_ESM.jpg]
